# Supplementary material for: Alignment between glioblastoma internal clock and environmental cues ameliorates survival in Drosophila
Source: Commun Biol. 2022 Jun 30;5:644. doi: 10.1038/s42003-022-03600-9 (PMC9247055; doi:10.1038/s42003-022-03600-9)
Supplement: Supplementary file 2 — Description of Additional Supplementary Files [file 42003_2022_3600_MOESM2_ESM.docx]

Description of Additional Supplementary Files

**File name:** Supplementary Data

**Description:** The source data behind the graphs in the paper.
