# Supplementary material for: Alignment between glioblastoma internal clock and environmental cues ameliorates survival in Drosophila
Source: Commun Biol. 2022 Jun 30;5:644. doi: 10.1038/s42003-022-03600-9 (PMC9247055; doi:10.1038/s42003-022-03600-9)
Supplement: Supplementary file 4 — Reporting Summary [file 42003_2022_3600_MOESM4_ESM.pdf]

nature portfolio

Corresponding author(s): Sergio Casas-Tinto, Francisco A Martin

Last updated by author(s): May 31, 2022

Reporting Summary

Nature Portfolio wishes to improve the reproducibility of the work that we publish. This form provides structure for consistency and transparency in reporting. For further information on Nature Portfolio policies, see our Editorial Policies and the Editorial Policy Checklist.

Statistics

For all statistical analyses, confirm that the following items are present in the figure legend, table legend, main text, or Methods section.

n/a	Confirmed	
☐	☒	The exact sample size (*n*) for each experimental group/condition, given as a discrete number and unit of measurement
☒	☐	A statement on whether measurements were taken from distinct samples or whether the same sample was measured repeatedly
☐	☒	The statistical test(s) used AND whether they are one- or two-sided<br>*Only common tests should be described solely by name; describe more complex techniques in the Methods section.*
☐	☒	A description of all covariates tested
☒	☐	A description of any assumptions or corrections, such as tests of normality and adjustment for multiple comparisons
☐	☒	A full description of the statistical parameters including central tendency (e.g. means) or other basic estimates (e.g. regression coefficient) AND variation (e.g. standard deviation) or associated estimates of uncertainty (e.g. confidence intervals)
☐	☒	For null hypothesis testing, the test statistic (e.g. *F*, *t*, *r*) with confidence intervals, effect sizes, degrees of freedom and *P* value noted<br>*Give P values as exact values whenever suitable.*
☒	☐	For Bayesian analysis, information on the choice of priors and Markov chain Monte Carlo settings
☒	☐	For hierarchical and complex designs, identification of the appropriate level for tests and full reporting of outcomes
☒	☐	Estimates of effect sizes (e.g. Cohen's *d*, Pearson's *r*), indicating how they were calculated

Our web collection on statistics for biologists contains articles on many of the points above.

Software and code

Policy information about availability of computer code

Data collection	*Provide a description of all commercial, open source and custom code used to collect the data in this study, specifying the version used OR state that no software was used.*
Data analysis	*Provide a description of all commercial, open source and custom code used to analyse the data in this study, specifying the version used OR state that no software was used.*

For manuscripts utilizing custom algorithms or software that are central to the research but not yet described in published literature, software must be made available to editors and reviewers. We strongly encourage code deposition in a community repository (e.g. GitHub). See the Nature Portfolio guidelines for submitting code & software for further information.

Data

Policy information about availability of data

All manuscripts must include a data availability statement. This statement should provide the following information, where applicable:

- Accession codes, unique identifiers, or web links for publicly available datasets
- A description of any restrictions on data availability
- For clinical datasets or third party data, please ensure that the statement adheres to our policy

The datasets generated during and/or analysed during the current study are available from the corresponding author.

Human research participants

Policy information about studies involving human research participants and Sex and Gender in Research.

Reporting on sex and gender	*Use the terms sex (biological attribute) and gender (shaped by social and cultural circumstances) carefully in order to avoid confusing both terms. Indicate if findings apply to only one sex or gender; describe whether sex and gender were considered in study design whether sex and/or gender was determined based on self-reporting or assigned and methods used. Provide in the source data disaggregated sex and gender data where this information has been collected, and consent has been obtained for sharing of individual-level data; provide overall numbers in this Reporting Summary. Please state if this information has not been collected. Report sex- and gender-based analyses where performed, justify reasons for lack of sex- and gender-based analysis.*
Population characteristics	*Describe the covariate-relevant population characteristics of the human research participants (e.g. age, genotypic information, past and current diagnosis and treatment categories). If you filled out the behavioural & social sciences study design questions and have nothing to add here, write "See above."*
Recruitment	*Describe how participants were recruited. Outline any potential self-selection bias or other biases that may be present and how these are likely to impact results.*
Ethics oversight	*Identify the organization(s) that approved the study protocol.*

Note that full information on the approval of the study protocol must also be provided in the manuscript.

Field-specific reporting

Please select the one below that is the best fit for your research. If you are not sure, read the appropriate sections before making your selection.

☒ Life sciences ☐ Behavioural & social sciences ☐ Ecological, evolutionary & environmental sciences

For a reference copy of the document with all sections, see nature.com/documents/nr-reporting-summary-flat.pdf

Life sciences study design

All studies must disclose on these points even when the disclosure is negative.

Sample size	The minimal sample size was 5 (only in one sample) or 6 (figure 2), although in the other figures the number is higher. However, this is clearly depicted in the graphs. The circadian analysis was initially performed in 32 flies, although some of them died during the experiment. Though, the minimal number of animals was over 21 with one exception (16 animals). In this case, analysis was performed only on one replicate. Regarding survival, at least 80 0 flies of each genotype were tested.
Data exclusions	No data were excluded from the analysis.
Replication	Most experiments were performed two times, rendering very similar results. Survival tests and figures 4 and 5 were replicated three times, also with very comparable results, so data were grouped and analyzed altogether. Figure 2 were performed only once, but blinding analysis was applied to avoid a possible bias (see below).
Randomization	All experiments were performed under controlled conditions of temperature and humidity in special incubators, although in different seasons.
Blinding	The analysis for figure 2 was performed blindly by two different people, without any previous knowledge of genotypes and type of experiment. The PAGE t-test was applied for each point, showing no statitistical significant difference.

Reporting for specific materials, systems and methods

We require information from authors about some types of materials, experimental systems and methods used in many studies. Here, indicate whether each material, system or method listed is relevant to your study. If you are not sure if a list item applies to your research, read the appropriate section before selecting a response.

Materials & experimental systems

n/a	Involved in the study
☐	☒ Antibodies
☒	☐ Eukaryotic cell lines
☒	☐ Palaeontology and archaeology
☐	☒ Animals and other organisms
☒	☐ Clinical data
☒	☐ Dual use research of concern

Methods

n/a	Involved in the study
☒	☐ ChIP-seq
☒	☐ Flow cytometry
☒	☐ MRI-based neuroimaging

Antibodies

Antibodies used	anti repo (DSHB), anti-nc82 (DSHB), anti HRP (Cell Signalling), anti PDF (DSHB), anti-GFP rabbit (DSHB), anti Elav (DSHB), Alexa 488, 647 (Life Technologies).
Validation	All of them are commercially available and control samples showed the correct staining as described both by the supplier and in other published manuscripts, not only from our group but also from other teams.

Animals and other research organisms

Policy information about studies involving animals; ARRIVE guidelines recommended for reporting animal research, and Sex and Gender in Research

Laboratory animals	Drosophila melanogaster. The stocks used from Bloomington Stock Center were: tub-Gal80ts (BL-7019), Repo-Gal4 (BL-7415), UAS-myr-RFP (BL-7119) UAS-LacZ (BL-8529), Elav-LexA (BL52676), pdf-LexA (a gift from F. Rouyer), UAS-dEGFR ; UAS-dp110CAAX (gift from R. Read, Read et al., 2009), LexAopRheb (gift from Nuria Romero), UAS-PI3K and UAS-GSK3 (gift from J. Botas). 1 to 4 days old male flies were collected for the experiments.
Wild animals	No wild type animals were involved
Reporting on sex	Only males were used in the experimental design
Field-collected samples	All fly stocks were maintained at 25ºC (unless otherwise specified) on a 12h:12h light:dark cycles at constant 60% of humidity in standard medium. Parental flies are maintained at 17ºC and selected flies were tranfered to 29ºC for 7 days or until death (in case of lifespan assays) at L:D 12h:12:, DD or L:D 14h:14h conditions.
Ethics oversight	No ethical approval was required, given the exclusive use of Drosophila for the experiments

Note that full information on the approval of the study protocol must also be provided in the manuscript.
